# Investigation of Recrystallization Kinetics in 1050 Al Alloy by Experimental Evidence and Modeling Approach

**DOI:** 10.3390/ma16175760

**Published:** 2023-08-23

**Authors:** Purnima Chakravarty, János György Bátorfi, Jurij J. Sidor

**Affiliations:** 1 Savaria Institute of Technology, Faculty of Informatics, Eötvös Loránd University (ELTE), Károlyi Gáspár tér 4, 9700 Szombathely, Hungary; 2 Doctoral School of Physics, Faculty of Natural Sciences, Eötvös Loránd University (ELTE), P.O. Box 32, 1518 Budapest, Hungary

**Keywords:** annealing, dislocations, stored energy, recrystallization, JMAK, 1050 Al alloy

## Abstract

The recrystallization (RX) kinetics of commercially pure Al alloy is studied under the scope of annealing temperature, time, and degree of deformation. To examine the distribution of recrystallization, Johnson–Mehl–Avrami–Kolmogorov (JMAK) theory is employed, where the path of microstructural transformation from the deformed state to the fully recovered one is studied as a function of the volume fraction of recrystallized grains ($X_{V}$) and annealing time. The drop in hardness is recorded for the samples at various stages of annealing with a corresponding decrease in stored energy as the annealing time increases. The stored energy obtained from the hardness results and Orientation Imaging Microscopy (OIM)-based method is found to be in good agreement with each other, proving the efficiency of both techniques. To determine the volume fraction of the recrystallized microstructure, data obtained from Vickers hardness measurements are used. Various parameters associated with recrystallization statistics such as the critical radius of nuclei, the incubation period, and the mobility of High-Angle Grain Boundaries (HAGB) were derived from the experimental evidence. The experimental data also suggest a sharp drop in the velocity of HAGB as the RX transformation process approaches its completion, which is found to be a direct result of a drop in stored energy. A softening window between 42 s and 55 s is identified for our experimental data where the hardness, stored energy, and velocity of HAGB drops very sharply, and the maximum fraction of deformed grains is expected to be converted to the recrystallized ones. Along with experimental observations, an analytical model was developed, which helps to approximate the kinetics of RX and corresponding parameters for various annealing temperatures and strains while revealing the characteristic feature of Avrami exponent *n*. Both experimental evidence and model data reveal a very strong dependency of recrystallization behavior on the stored energy.

## 1. Introduction

The optimum properties of metal alloys are broadly controlled by consecutive deformation and heat treatment techniques, in both commercial platforms and laboratory environments. In the case of highly anisotropic metals such as aluminum (Al), to improve the deep drawability properties, the ability to control the microstructure and texture of the final product is of critical importance. Although numerous investigations have been conducted in this area [1,2,3], a fundamental understanding and quantification of the relative parameters are significant in the field of research on aluminum for successful industrial implementation.

The control of microstructure via heat treatment procedure greatly depends on the mechanism of recrystallization in metals. The phenomenon of recrystallization (RX) is defined as the process of consumption of deformed grains and their replacement with the new stress-free domains via the formation and migration of High-Angle Grain Boundaries (HAGB) during heat treatment at RX temperature [4]. The physical process of the complete transformation of deformed grains to recrystallized ones is achieved via recovery, primary RX, and secondary RX in some instances, as described elsewhere [4,5]. The focus of the current scientific contribution is the primary RX phenomenon under the scope of both qualitative and quantitative analysis. It is important to underline that, in the present investigation, the term “*transformation*” will be related to recrystallization, which refers to the replacement of deformed grains by recrystallized ones.

The process of recrystallization can restore the microstructure of a deformed metal to a lower energy level, which is typically elevated during cold working due to the presence of dislocations. One of the most important aspects of RX is the manipulation of microstructure by controlling the size and respective orientation of the grains. An appropriate fusion of proper microstructural and textural features is desirable for reaching the necessary mechanical behavior in Al alloys. To design the desired RX microstructure, not only grain refinement but also texture control and the release of stored energy are of importance [2,4]. However, the kinetics of recrystallization is critical to the various physical elements mentioned above when it comes to controlling the microstructure. The physical aspects of RX kinetics are a complex subject of study and have a tight connection with factors such as (a) the deformed state of the material, (b) the amount of alloying elements, (c) the annealing temperature, (d) the annealing environment, (e) the duration of annealing, etc. The kinetics of RX is traditionally studied by the Johnson–Mehl–Avrami–Kolmogorov (JMAK) Equation [6,7,8,9,10]:

(1) $$X_{V}=1-e^{-Bt^{n}},$$

where $X_{V}$ is the recrystallized fraction of the microstructure, *t* is an annealing time, *n* is the Avrami exponent, and *B* is a constant. Factor *B* reflects material properties at a certain annealing temperature *T*, while *n* imposes the effect of annealing time on RX kinetics [4,6,7,8]. Since Equation (1) is an Arrhenius-type equation, changes in annealing temperature tend to greatly affect the magnitude of the constant *B*, and, mathematically, *B* is expressed as [4,6,7,8]:

(2) $$B=\frac{f{NG_{v}}^{3}}{4},$$

In Equation (2), *N* is the nucleation rate of new grains, $G_{v}\;$is the growth rate of RX grain, and *f* is the shape factor associated with the shape of nucleated grains, which is given by 4π/3. There are two possible limiting cases of nucleation rate *N* that influence the numerical value of the Avrami exponent *n*:

- The first case is when the nucleation rate remains constant throughout the microstructural transformation process (*n* = 4) [6].
- The second possible scenario is the site-saturated nucleation, i.e., most of the nucleation events tend to take place at the beginning of the recrystallization process, where *n* is supposed to be below 4 [6,7,8]. However, various literature sources suggest that, for site-saturated nucleation, the value of *n* is below 3 [11,12].

The nucleation rate N of the RX transformation is derived from the number of nuclei generated per volume within a unit of time as the annealing procedure starts. The major nucleation sites are reported to be as follows [2,3,13,14,15,16]:

- Deformed zones around non-deformable particles i.e., the Particle-Stimulated Nucleation (PSN) mechanism of RX.
- Cube grains present in the material prior to TMP and subjected to deformation are responsible for the nucleation of new Cube grains.
- Grain boundary.
- Other deformation hot zones, such as shear bands, deformation bands, and regions with high deformation inhomogeneity.

On the other hand, irrespective of the source of nucleation, the number of nuclei generated per unit volume in the deformed matrix per second (*N*) can be estimated as follows [6,7,8,17]:

(3) $$N=N_{0}\;e\;\exp\left(-\frac{\gamma_{g}b^{2}}{k_{B}T}\;\right),$$

In Equation (3), *N*_0_ is a model parameter, *e* equivalent strain, *γ_g_* surface energy of the grain boundaries, *b* is the Burgers vector, *k_B_* Boltzmann constant, and *T* is the annealing temperature. However, for an embryo or a sub-grain to convert to a viable nucleus, that is, to be able to grow, it must have a larger radius compared to the critical value ($r_{c})$ [4,18]:

(4) $$r_{c}=\frac{2\gamma_{g}}{E_{D}}\;,$$

Here, *E_D_* is the stored energy of the material. 

It must be noted that *E_D_* and $r_{c}$ are functions of annealing time [18]. The term stored energy has a special significance in the study of recrystallization phenomena, which will be discussed in the context of the growth rate $G_{v}$. Since the nucleation of new grains is not enough for the progress of RX, the high-angle grain boundaries (GB) must grow. The process of growth is associated with the velocity of the high-angle grain boundaries, which is equivalent to the growth rate $\left(G_{v}\right)$ and is expressed as follows [4,19]:

(5) $$G_{v}=M_{GB}E_{D},\;$$

Here, $M_{GB}$ is the mobility of the high-angle grain boundaries (HAGB), which varies with temperature. The above-mentioned stored energy (*E_D_*) is the main driving pressure created at GB during recrystallization. During straining, the material goes through plastic deformation, which changes the size, shape, and crystallographic orientation of the grains. Along with that, deformation induces a large number of linear defects in the material. Apart from making microscopic changes, a portion of the energy accumulated during the deformation process remains trapped in the material (*E_D_*) in the form of dislocations [20]. Hence, stored energy (or residual energy of dislocations) can be expressed with the help of dislocation density [4]:

(6) $$E_{D}=\alpha \rho \;Gb^{2},$$

where *G* is the shear modulus, *α* is a geometric constant, and ρ is the dislocation density. 

Hence, apart from ρ, all other parameters are tagged to the primary characteristics of the material under study. It is well-known that plastic deformation takes place by the generation, motion, subsequent trapping, and accumulation of dislocations, thereby increasing the overall dislocation density [4,20]. This implies that the driving force for recrystallization can be controlled during the mechanical processing of the material. It must be noted that the hot deformation process can lower the overall driving force as recovery, and the annihilation of dislocations takes place at elevated temperatures, although an accountable amount of deformation is achieved [21].

The mobility of HAGB $M_{GB}$ is not an intrinsic property of a parent crystal but changes significantly depending on the alloying element and its amount in the metal alloy. The $M_{GB}$ has also a definite effect on the development of recrystallization textures [3,19]. However, the quantification of this parameter and establishing an experimental method are equally important. Generally, $M_{GB}$ is expressed as follows [4]:

(7) $$M_{GB}=M_{0\;}\exp\left(-\frac{Q_{G}}{k_{B\;}T}\right),$$

where *M*_0_ is a material constant, and *Q_G_* is the activation energy required for the migration of the HAGB [19].

The present scientific work intends to evaluate the kinetics of recrystallization by both experimental and modeling techniques. When it comes to the modeling of recrystallization phenomenon, various established algorithms can be found in the literature, such as Cellular Automata, Continuum Mechanical Models, Monte Carlo Potts models, Phase Field Models etc. [3,22,23,24,25,26,27]. However, these simulation methods are complex in nature and time-consuming. At the same time, these numerical approaches cannot predict all the parameters associated with RX. The model presented in the given research work depends on only one quantity, which is the driving force of recrystallization provided by the density of dislocations. In the modeling approach, the stored energy is estimated from the dislocation density estimated by the Kubin–Estrin Model [28,29,30]. A detailed explanation of the modeling approach of RX kinetics is provided below. 

The driving force of RX is experimentally obtained from Vickers hardness measurements and the OIM method in a comparative manner. From the respective fraction of the experimentally obtained recrystallization, corresponding RX parameters are quantified. Along with that, an analytical technique has been developed, enabling the investigation of recrystallization kinetics at different temperatures and strains. The algorithm discussed in the frame of this study can be applied to an arbitrary metal using proper material constants. The majority of physical quantities are measured experimentally and computed by various numerical approximations. It can be shown that the computed and measured quantities are comparable to each other and fully versed in the wide range of corresponding experimental evidence.

## 2. Experimental Procedure 

**Figure 1:**
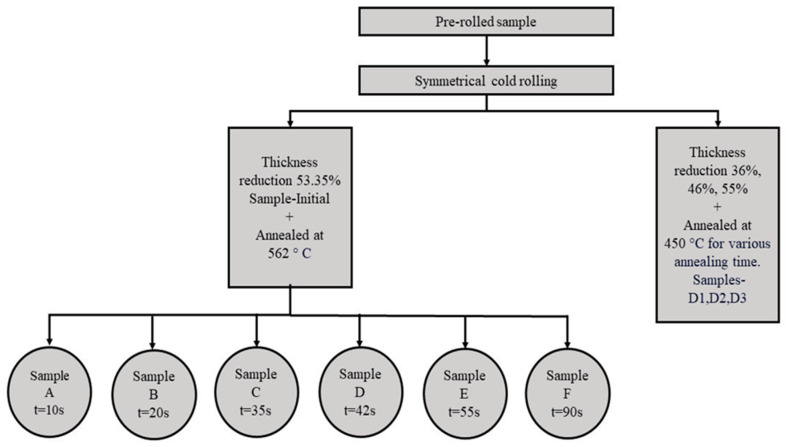
TMP path of the investigated 1050 aluminum alloy.

The alloy under investigation is 1050 Al, a commercially pure alloy with ~0.3 wt% of Fe. The chemical composition of the chosen alloy comprises minimal solute content, which makes it one of the most fundamental Al systems, and hence ideal for elemental study. The sheet material was subjected to full recrystallization by annealing at 550 °C for 40 min to eliminate any stress from the manufacturing process (virgin sample). After heat treatment, the sample was subjected to symmetric cold-rolling with thickness reductions of 53.35% (Sample Initial) by a laboratory-rolling machine with a roll diameter of 150 mm. The performed rolling was a single pass non-lubricant rolling. After deformation, the sample was annealed to study the RX kinetics. The deformed sample was furnace annealed at 562 °C for 10 s (sample A), 20 s (sample B), 35 s (sample C), 42 s (sample D), 55 s (sample E) and 90 s (sample F), followed by quick water quenching. Prior to annealing experiments, the Nabertherm box-type furnace was pre-heated to 562 °C and was held at this temperature for 30 min before placing the sample inside. The thermomechanical processing life cycle of the material under study is presented in Figure 1.

The virgin material was additionally subjected to 36%, 46%, and 55% thickness reductions and annealed at 450 °C for various times, followed by quick water quenching, with the aim of verifying the numerical approach employed for the simulation of recrystallization kinetics.

To estimate both the softening of the deformed material during annealing and the hardness values of the samples, microhardness tests were performed. The indentation data were recorded using the Zwick/Roell^®^ ZHVμ-type Vickers microhardness tester (ZwickRoell, Brierley Hill, West Midlands, UK) by making diamond shape indents on the Transverse Direction (TD) plane of investigated samples. Noting the consequences of the Indentation Size Effect (ISE) [31], the indentation was performed for loads falling in the saturation zone of the ISE curve. In the present case, a load of 2 kgf (19.62 N) was employed, so the hardness impact from the maximum number of grains was recorded. All samples subjected to indentation were prepared under the guidelines of the standard sample preparation technique, which consists of mechanical grinding and polishing. During the process of mechanical polishing, Struers^®^-type DiaDuo suspensions with 3 μm and 1 μm diamond particles were used. The mechanical polishing of the samples was followed by soap cleaning under running water and drying using a blow-drier.

For the microstructural characterization, the samples were exposed to Electron Back-Scattering Diffraction (EBSD) scans across the thickness of the TD plane. To make the samples ready for the scan, the mechanically polished samples were electropolished. The electrolytic polishing was performed using a Struers^®^ A2 electrolyte that was pre-cooled (0–5 °C). During polishing for 45–60 s, the voltage was maintained in the range from 20 V to 30 V. The EBSD camera (Hikari-type^®^ detector, EDAX Inc., Mahwah, NJ, USA) was attached to a high-resolution Scanning Electron Microscope (SEM) FEI-Teneo equipped with an FEG filament (Thermo Fisher Scientific, Brno, Czech Republic). The EBSD scanning was performed twice for each sample: first for the microstructure and texture investigation (large areas with step sizes ranging between 1–5 μm), and then for the assessment of Geometrically Necessary Dislocations (GND) (relatively small areas with step sizes of ~0.5 μm). The GND scans were performed for the selected samples only because of time-consuming measurements. During the GND scans, a very slow data acquisition rate (~10 frames per second (fpc) or even lower) was used. However, an acquisition speed of ~20 fpc or even higher was employed for the texture assessment. The accelerating voltage maintained during Orientation Imaging Microscopy (OIM) was about ~17–19 kV to capture the patterns efficiently. The tilting of the sample inside the SEM chamber was 70° with respect to the EBSD detector. The EBSD scans were performed on the hexagonal grids.

For the post-processing of OIM data, the commercial OIM-TSL-8^®^ software (EDAX Inc., Mahwah, NJ, USA) was used. The calculated Orientation Distribution Functions (ODFs) are displayed for the φ_2_ = 45°, φ_2_ = 65° and φ_2_ = 90° sections. These three ODF sections tend to reveal the most important texture components and corresponding fibers.

## 3. Results and Data Analysis

The influence of both pre-rolling and deformed states on the RX kinetics is immense, as the RX grains grow at the expense of energy, stored in the deformed material in the form of dislocations [2]. In cases where the material is subjected to straining before rolling, the dislocation density will increase even further during cold-rolling. Consequently, capturing the impact of deformation on the RX state becomes challenging, and therefore it is important to inspect both the pre-rolled and deformed samples.

### 3.1. Pre-Rolling State 

**Figure 2:**
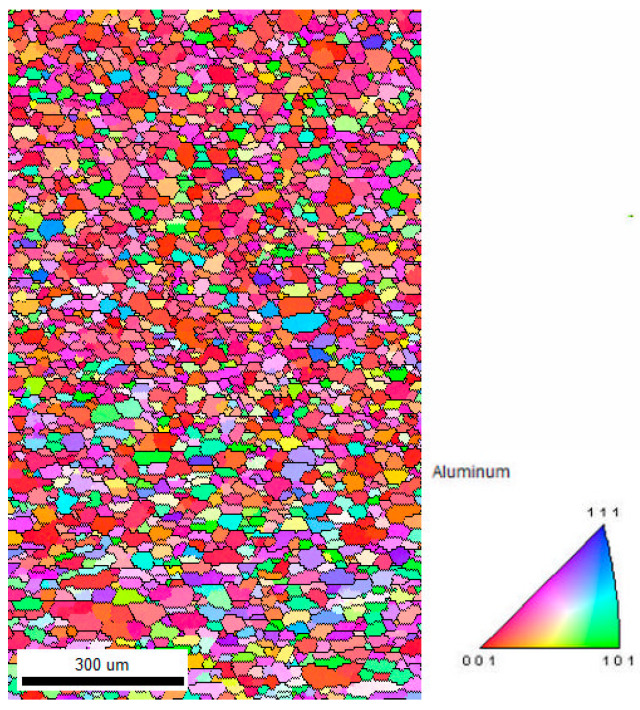
IPF map of the pre-rolled sample.

**Figure 3:**
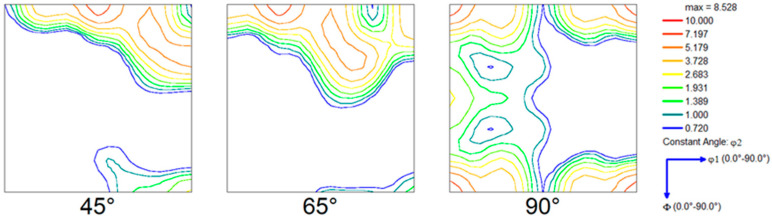
Texture evolved in the investigated pre-rolled sample.

The as-received material was heat-treated to ensure the stress-free microstructure prior to deformation (virgin sample). A hardness of 2.15 × 10^8^ Pa was recorded for this sample. The corresponding microstructure is presented with the help of the Normal Direction Inverse Pole Figure (IPF) map in Figure 2, while Figure 3 shows the corresponding crystallographic texture.

It can be seen from Figure 2 that the microstructure of the pre-rolled sample shows negligible orientation contrast deviation in each grain, implying that the material is fully recrystallized and “free” of dislocations (one should note that even the fully recrystallized material contains a relatively large amount of dislocation, typically ~10^10^ m^−2^). The dominating red color on the IPF map suggests that a {001}//ND-dominating texture evolved. The ODF map of Figure 3 reveals the quantitative aspect of texture evolution in the pre-rolled sample. The ODF is dominated by a strong Cube texture, mixed with the rotated cube texture components aligned along the {001}//ND fiber and weakly developed Goss orientation.

### 3.2. Deformed State

**Figure 4:**
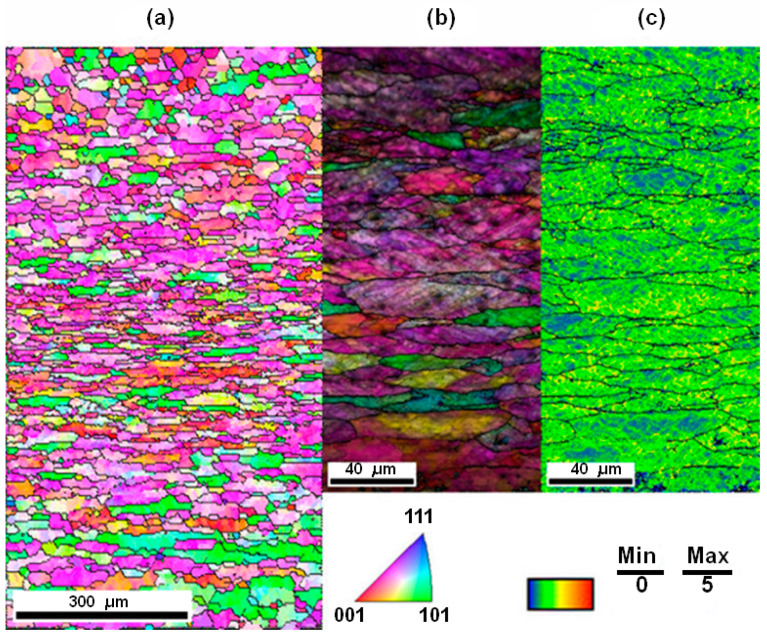
(**a**) IPF map, (**b**) IQ+IPF map and (**c**) KAM map of deformed sample (initial).

As reported by Cahn, nucleation occurs in the locally recovered region that already exists in the heterogeneously deformed microstructure, characterized by dislocation-rich zones [32,33]. Therefore, to ensure fertile or stable nucleation, the heat-treated virgin sample was cold-rolled with a 53.35% thickness reduction. The microstructure of Figure 4a shows that the effect of deformation on the virgin sample is prominent, as the highly misoriented grains are aligned with the rolling direction (RD). The grain size for the deformed sample is recorded as 29.8 µm. Since the deformation tends to produce dislocations, and the density of dislocations in the deformed material has great importance in RX, the deformation structure was examined by constructing a Kernel Average Misorienting (KAM) map (Figure 4c) as well as by an IPF map superimposed with the Image Quality (IQ+IPF) map (Figure 4b). The KAM map was calculated with a threshold misorientation of 5° to capture the dislocation substructures proficiently, namely Geometrically Necessary Dislocations (GND) [34]. In Figure 4b,c, both IQ+IPF and KAM maps display complex dislocation structures developed in the initial sample as an impact of the deformation process. The dislocations are distributed non-uniformly throughout the microstructure with high- and low-density regions of dislocation substructures (KAM maps). In addition, the dislocation-rich regions form complex structures, such as deformation bands or cell structures, as shown in Figure 4b (IQ+IPF).

A qualitative study of the deformation substructure suggests an increase in the extent of micro-structural misorientations, as well as the total dislocation density (*ρ*) of the material, whose direct impact can be investigated by hardness testing since an increase in *ρ* can impart strain-hardening [4]. The Vickers hardness testing reveals that hardness increased from 2.15 × 10^8^ Pa (pre-rolled sample) to 4.507 × 10^8^ Pa (deformed sample with 53.35% reduction). The dislocation density for the deformed state is estimated from the hardness values (*H_v_*) using the following relation [35]:

(8) $$\rho ={\frac{1}{\alpha^{3}}\left(\frac{H_{v}}{3.06MGb}\right)}^{2},$$

In Equation (8), *M* is the Taylor factor. The value of *α* calculated for the deformed sample with a Von Mises equivalent strain (*e*) of 0.88 is 0.5612 (*α* is calculated according to the procedure described in ref. [35]). The ultimate dislocation density calculated by Equation (8) is 2.03 × $10^{14}$ m^−2^. The order of computed dislocation density falls in the range of experimentally observed and calculated values for Al1050 alloy [35].

### 3.3. Release of Stored Energy during Annealing 

**Figure 5:**
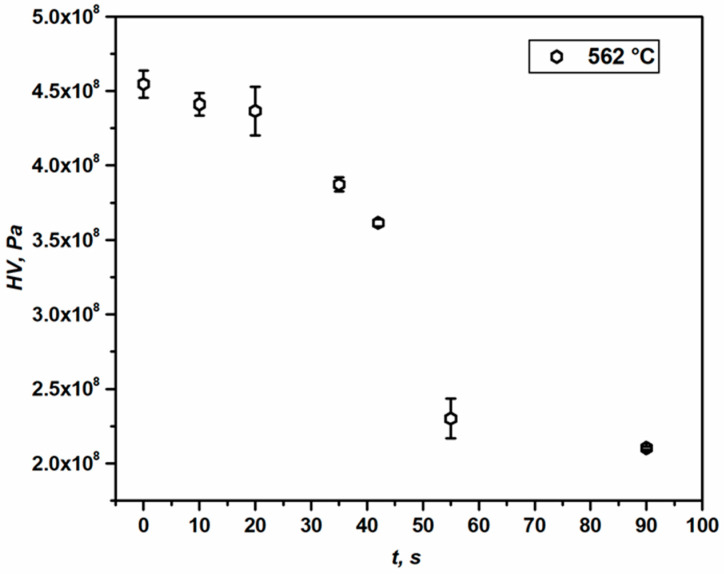
Softening behavior of the deformed aluminum 1050 alloy annealed at 562 °C: the Vickers hardness as a function of annealing time *t*.

As the process of annealing proceeds, the complex dislocation structure formed during cold-rolling tends to dissolve to a certain extent by the annihilation of dislocations. The annealing phenomena taking place on the sub-structural level tend to decrease the total dislocation density in the material; hence, the hardness is also expected to fall. This phenomenon is known as material softening [4]. Keeping note of the nature of RX evolution, the Vickers hardness (*HV*) measurement was recorded with particular care since microstructure development is highly inhomogeneous [21]. The load of 2 kgf was used to impact a large area and avoid a very local hardness measurement. The number of indentation measurements (~5–10) provided reliable statistics. The softening curve for the sample under consideration is presented in Figure 5. The annealing of the deformed sample (see Figure 1) was performed at 562 °C for 10 s (sample A), 20 s (sample B), 35 s (sample C), 42 s (sample D), 55 s (sample E), 90 s (sample F) and the hardness for the annealing time of 0 s stands for the initial sample. It is obvious from Figure 5 that with an increase in annealing time, the hardness tends to decline on average, and a sharp drop in *HV* between 42 s and 55 s can be noticed. This drastic decrease in hardness can be an indication of significant softening. After 55 s of annealing, the softening rate tends to level off, with a hardness of 2.1 × 10^8^ Pa (sample F).

To further understand the softening phenomenon, we can define the stored energy (*E_D_*) in terms of hardness using Equations (6) and (9) [35,36]. Alternatively, special care must be taken while estimating the dislocation density of annealed material using hardness values, as these calculations can be affected by the Hall–Petch relationship [37]. Equation (8) enables the calculation of dislocation density based on hardness values. When the sample is deformed, the dislocations accommodate themselves in the material due to the applied strain. In both moderately and highly deformed samples, the hardness values are majorly affected by the dislocation density, and the contribution of *ρ* to *H* is orders of magnitude higher than the grain boundary strengthening or that provided by the impurity of atoms [35,38]. However, the same is not true for annealed or slightly strained materials [39]. In this case, the density of dislocation is low, and the overall hardness value is influenced by the effect of other factors, such as grain boundary strengthening or the presence of impurities, along with the effect of dislocations. By using Equation (8), we intend to capture the effect of dislocation density only. From the perspective of the current scenario, it must be noted that the alloy under consideration (1050 Al alloy) is of high purity and ensures a single-phase state with a minimum number of second phases. In view of this, it is reasonable to assume that the grain boundary strengthening majorly influenced the hardness values, especially in fully annealed material. The hardness of fully annealed material is denoted by *H*_0_. Hence, to effectively account for the contribution of dislocation density on the hardness values, *H*_0_ is subtracted from the instantaneous hardness *H_v_* of the annealed (partially recrystallized) samples. As mentioned elsewhere [40,41], cold work can increase the dislocation density to 10^16^ m^−2^ from approximately 10^10^–10^12^ m^−2^ in unstrained metals. Hence, the annealed samples must contain at least the minimum dislocation density, accommodated by the sample in its unstrained or fully recrystallized state, given by *ρ*_0_. Given the above, Equation (8) gains the following form:

(9) $$\rho =\rho_{0}+{\frac{1}{\alpha^{3}}\left(\frac{H_{v}-H_{0}}{3.06MGb}\right)}^{2},$$

where *ρ*_0_ = 10^10^ × 1/m^2^ [4,26]. 

**Figure 6:**
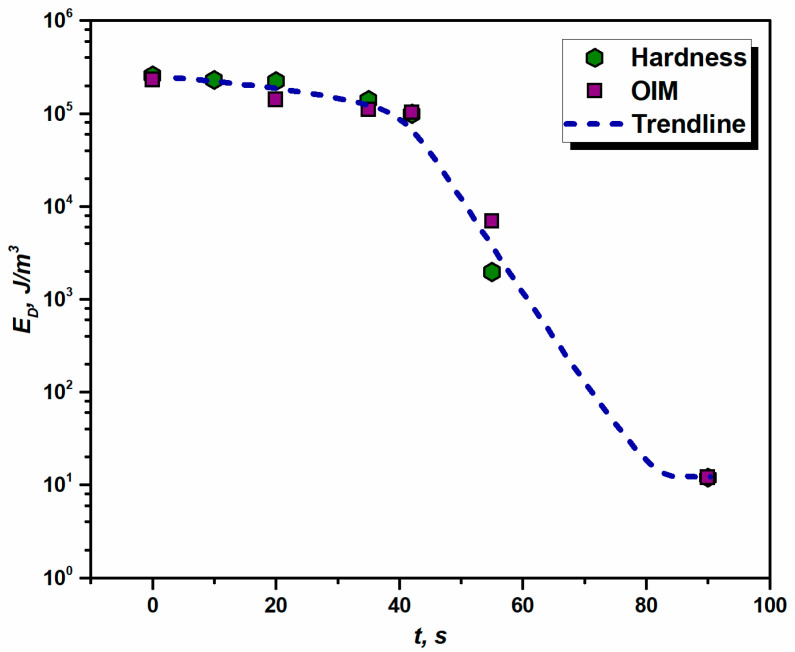
Release of stored energy with annealing time *t* in 1050 aluminum alloy annealed at 562 °C.

Using Equation (6), the stored energy of dislocations (*E_D_*) at each stage of annealing has been calculated. The calculated *E_D_* values from hardness are presented in Figure 6. The residual energy of dislocations calculated by Equations (9), (8) and (6) is a direct impact of the recorded hardness for both deformed and annealed samples. While calculating *E_D_* using Equation (6) for sample A, the Taylor factor used is 3.24, equal to the deformed sample (Initial) (Table 1), as the difference in hardness between these two samples is very small (Figure 5) and the effect of annealing on sample A is considered to be trivial. The dislocation density is approximated under the consideration that the total dislocation density is about twice the density of GND, as per Ref. [42]. Using this approach, it is assumed that the overall dislocation density is made up of both Statistically Stored Dislocations (SSD) and GNDs contributing 50% each to the total ρ; which is experimentally proven [42].

The GND is calculated by using Kamaya’s technique [43] and special care was taken while choosing the step size, as noted in the experimental procedure, to reduce the level of noise in the recorded data. In Kamaya’s approach, the gradient of KAM misorientations, calculated from the 1st to the 10th neighbor (depending on the EBSD step size), is used to estimate the GND density in respective samples [42,43]. The total dislocation density for sample F is approximated to be 10^10^ m^−2^, which stands as the dislocation density for unstrained material [35], as the hardness of the pre-rolled material and sample F were found to be comparable. Taylor factor (*M*), obtained from the EBSD scans, is presented in Table 1, together with the GND densities. The change in the residual energy of dislocations *E_D_* with annealing time, derived from the GND, is presented in Figure 6 under the OIM section.

The values of *E_D_* estimated by hardness and OIM measurements (see Figure 6) follow a declining trend (see the dashed trendline) and clearly show that the driving force for recrystallization in the advanced stages of annealing is negligible.

### 3.4. Microstructure and Texture Evolution during Annealing

**Figure 7:**
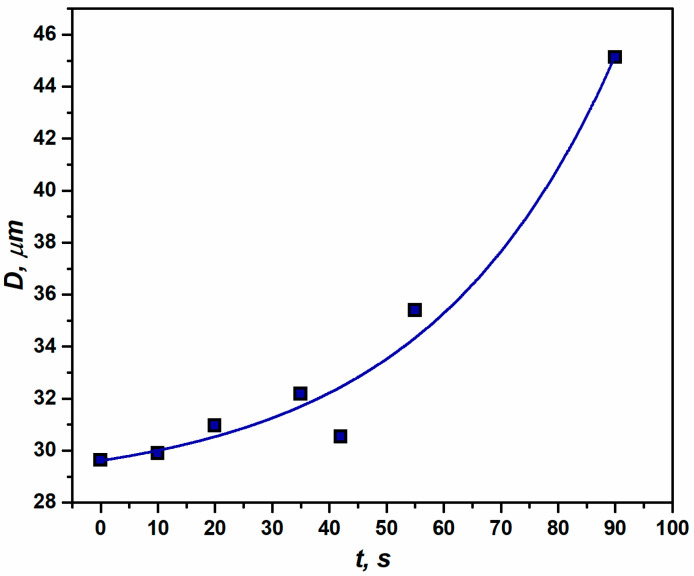
Effect of annealing time on the average grain size in aluminum 1050 alloy at *T* = 562 °C.

The overall grain size (*D*) is found to increase throughout the process of annealing from the deformed state of the material, as presented in Figure 7. However, the dependence of *D*(*t*) shows different stages of the RX process in terms of kinetics. In the beginning, a sluggish and relatively steady phase of grain growth is observed up to 55 s, while the remaining part points toward the drastic grain-coarsening (between 55 s (sample E) to 90 s (sample F)). The type of grain distribution that was observed is a characteristic feature of metals with cubic lattices [44]. The RX grain size depends on various technological factors. For instance, a high amount of applied strain results in a fine grain size since it favors a rapid nucleation rate, caused by the large number of nuclei [4].

**Figure 8:**
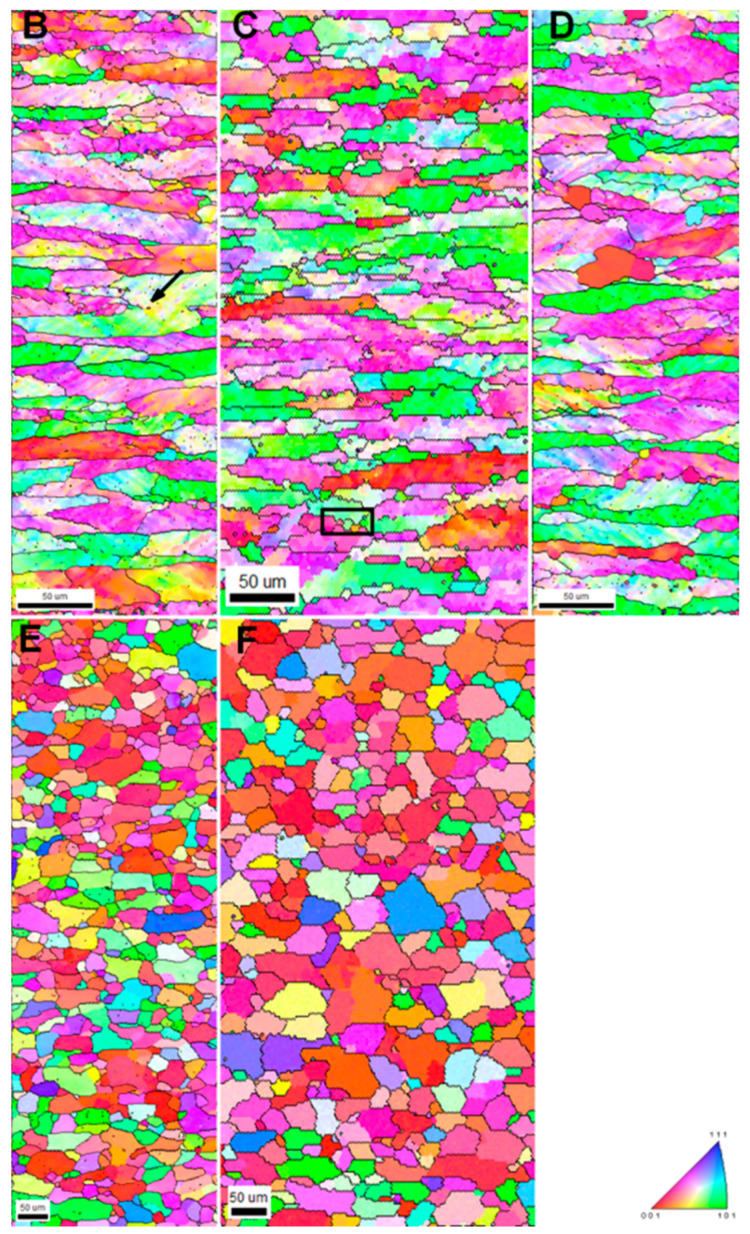
Evolution of microstructure in aluminum 1050 alloy annealed at 562 °C. IPF map of samples (B–F); the labels refer to sample name.

To acquire a better understanding of the annealing phenomena, the IPF maps for the selected samples are presented in Figure 8. Figure 8 clearly depicts the transformation of strained grains (see Figure 8B–E) to fully recrystallized counterparts (Figure 8F, sample F). In the early stages of annealing, a large amount of recovery is expected to take place, because of the nucleation of new stress-free grain that evolved from the deformed microstructure [4]. Small RX grains are found to be nucleated around the high-angle grain boundaries during the microstructural transformation (marked by a black box). In the case of sample D, which is annealed for 42 s at the temperature of 562 °C, an increase in RX grain size can be seen. Similarly, for sample E (55 s holding time), a large number of RX grains are visible, which are converted to fully recrystallized microstructure in 90 s (sample F). Although some low-angle misorientations are still present within the RX grains, they can be considered one of the characteristic features of the RX microstructure. At the beginning of RX, signs of the consumption of high-angle grain boundaries were observed (marked with arrow sign, Figure 8B, sample B) in numerous instances. 

**Figure 9:**
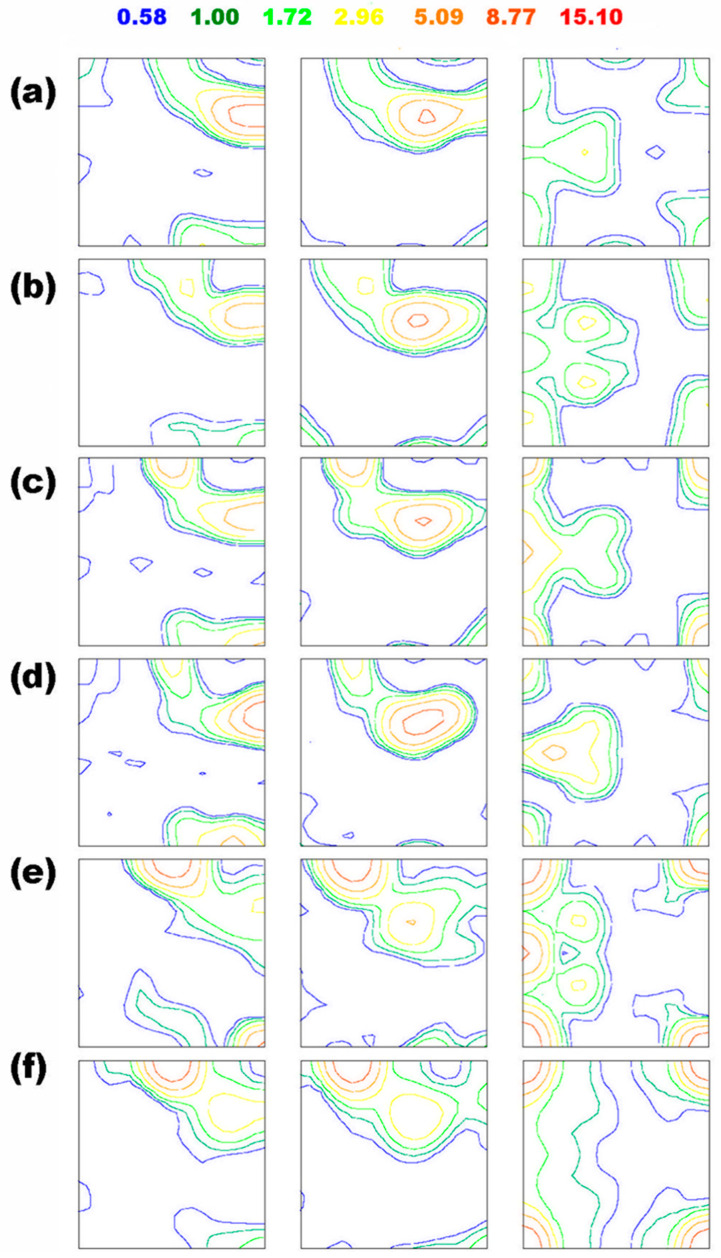
Three ODF sections for (**a**) initial sample, ODF_max_ = 11.91; (**b**) sample B, ODF_max_ = 9.8; (**c**) sample C, ODF_max_ = 9.5; (**d**) sample D, ODF_max_ = 13.36; (**e**) sample E, ODF_max_ = 13.61; and (**f**) sample F, ODF_max_ = 15.05.

Throughout the process of recovery and recrystallization, a significant change in the orientation of the grains was noticed. To obtain a more in-depth understanding of the texture formation during the RX process, ODFs were analyzed at various stages of recrystallization (Figure 9). A comparative study of the texture of the virgin (pre-rolling state) sample (Figure 3) and initial sample (deformed) (Figure 9a), reveals that the Cube-dominating texture of the virgin sample rotated towards the β-fiber in the case of the initial sample. The texture present in the deformed material also comprises a very weak Cube and H component (a 45° rotated Cube orientation). The β-fiber, which evolved in the deformed material, is one of the very typical rolling textures identified by a complex-shaped fiber that connects the Copper {112}<111> and Brass {011}<211> components via {123}<9 15 11>, {314}<5 9 6>, and other components in the Euler space [45,46]. The annealing procedure was performed at 562 °C, and as the holding time increases, the rotation of deformation texture components towards the annealing texture becomes clearly visible, as shown in Figure 9b–f. 

For the annealing time of 20 s (sample B) the Copper and {123}<9 15 11> components become weaker compared to the deformed state; however, the Cube orientation dominates. It has been observed that the {123}<9 15 11> component tends to weaken with the progress of RX. The characteristic RX texture, i.e., the Cube component becomes dominant as the annealing time reaches 90 s (sample F). Samples E and F have a weak H {001}<110> orientation, along with other RX textures. Out of all annealed samples, sample F was found to comprise the highest texture intensity of 15.05 with a maximum at {100}<001>. The path of texture transformation shown in Figure 9 is typically observed in FCC materials of high stacking fault energy [4,47]. The replacement of rolling texture with the RX one causes a drop in the Taylor factor (see Table 1), since the *M* values of the β-fiber are higher compared to the RX texture components, such as the Cube, rotated cube, or Goss orientation [3]. Inasmuch as the Taylor factor shows a degree of dissipated plastic power in a particular orientation, the average *M*, calculated for the polycrystalline aggregate, can be correlated with the stored energy, although the correlation is of a qualitative nature. This type of approximation led to the successful modeling of texture evolution in various Al alloys after different straining levels [3,16]. In light of the concept discussed, one can conclude that the high-stored energy domains are replaced by the low-stored energy ones, which supports the hypothesis of low-stored energy nucleation [3,4,16] in FCC metals.

### 3.5. Study of RX Kinetics from Hardness

The study of recrystallization phenomena is strongly dependent on the quantification of the recrystallized fraction of microstructure $X_{V}.$ In the present contribution, the fraction of recrystallized volume is estimated from Vickers microhardness measurements. 

While calculating$\;X_{V}$, the RX fraction is associated with the softening effect, accompanied by recovery, recrystallization, and grain growth due to annealing. The kinetics of RX for the annealed samples are analyzed using Equation (10) [48]:

(10) $$X_{V}=\frac{H_{m}-H_{v}}{H_{m}-H_{0}},$$

**Figure 10:**
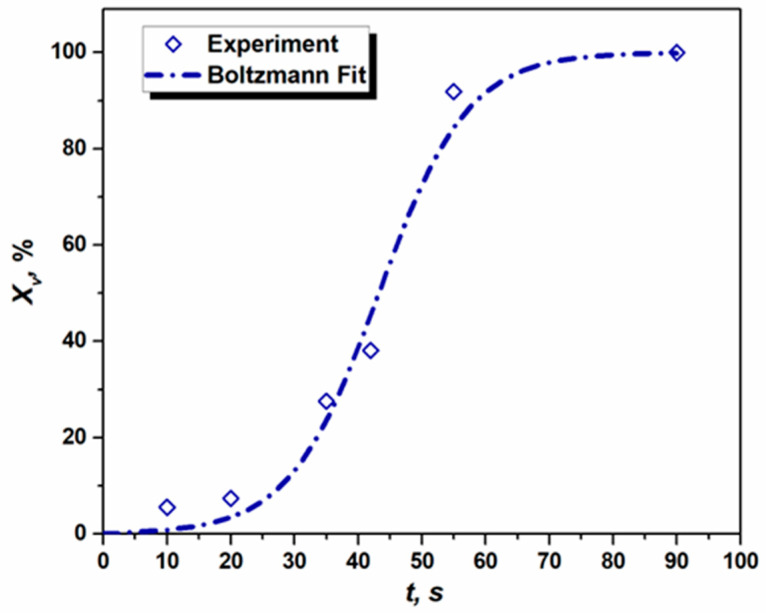
Kinetics of recrystallization (*T* = 562 °C) measured by indentation in 1050 Al alloy (The dash-dotted line represents Boltzmann fit).

Here, *H_m_* and *H*_0_ are the hardness of the sample in the deformed state (initial sample) and fully annealed state (sample F). The *H_v_* is the instantaneous hardness of the samples annealed for different *t*. While calculating the fraction of recrystallized grains using Equation (10), the hardness values presented in Figure 5 were used. The dependence of $X_{V}$ on annealing time is presented in Figure 10 for samples A–F. 

### 3.6. Quantification of Parameters Associated with the Kinetics of RX from Experimental Evidence

While quantifying parameters associated with the kinetics of recrystallization, it is important to define the constant *B* in the JMAK Equation. By fitting Boltzmann function to the distribution of $X_{V}$ (in %) vs. annealing time *t*, it is possible to extract the half-time for the completion of recrystallization:

(11) $$X_{V}=100-\frac{100}{1+e^{(t-t_{0.5})/dt}},$$

where *t*_0.5_ is the half-time of recrystallization (time required to ensure $X_{V}$ = 50%), *t* and *dt* are the instantaneous annealing time and time increment, respectively.

The advantage of using the Boltzmann distribution is that one can directly derive the half-time (*t*_0.5_) of RX from the experimentally measured dataset without knowing *B* and *n*, which is necessary for the JMAK Equation. In the present case *t*_0.5_ = 43.2 s, which can be seen in Figure 10. 

**Figure 11:**
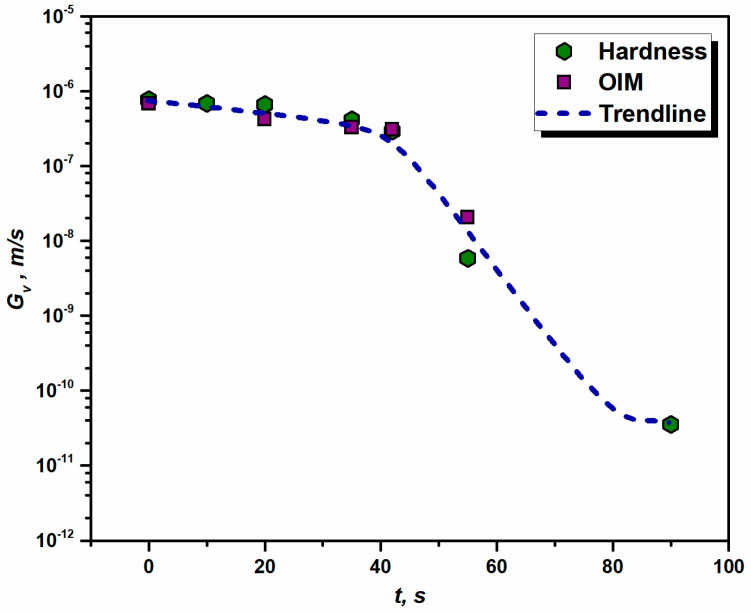
Effect of annealing on the velocity of high-angle grain boundaries in aluminum 1050 alloy with an increase in holding time (*T* = 562 °C, *e* = 0.88).

The mobility of HAGB, $M_{GB}$ for aluminum 1050 annealed at 562 °C is estimated using Equation (7). The value of activation energy, *Q_G_* = 8 × 10^−20^ J and *M*_0_ is a material constant equal to 3.1 × 10^−9^ m^4^J^−1^s^−1^ and Boltzmann constant, *k_B_* = 1.38 × 10^−23^ JK^−1^ is considered for further calculations, as per Refs. [17,49]. The estimated mobility value for HAGB at 562 °C is $M_{GB}$ = 2.98 × 10^−12^ m^4^J^−1^s^−1^. To further calculate the velocity of HAGBs $\left(G_{v}\right)$ one can employ Equation (5), where $G_{v}$ is a function of stored energy (*E_D_*) and temperature-dependent term $M_{GB}$. The calculated values of $G_{v}$ are presented in Figure 11, which indicates that the velocity of HAGB decreases drastically ($G_{v}$ drops by four orders of magnitude) as the annealing time increases, implying that the motion of HAGBs tends to slow down as the transformation in the microstructure takes place. Figure 11 suggests that, after 50 s of annealing at 562 °C, the grain boundary motion is negligible.

Another significant parameter in evaluating RX kinetics is the occurrence of nucleation. However, for viable nucleation to take place, the annealing time must exceed the incubation period and subgrains should have a larger radius than the critical one [4]. The incubation period (∆t) is estimated by the relationship [50]:

(12) $$∆t=\frac{2\gamma_{g}}{M_{GB}{\;E}_{D}^{2}{\;\chi }_{max}}-\frac{r_{0}}{M_{GB}\;E_{D}},$$

In Equation (12), $E_{D}$ stands for the stored energy of a material in the deformed state ($E_{D}$, obtained from both hardness and OIM can be used; in the present case, hardness data are used due to their simplicity in the experimental procedure). The $r_{0}$ ≈ 0.25 µm is the average radius of subgrains [50,51] in the early stage and $\chi_{max}$ ≈ 2.5 is the normalized value for the largest subgrain in a given microstructure distribution [50]. The energy of GB (*γ_g_*) should be estimated to define the Δ*t*. The value of *γ_g_* can be estimated by the Read and Shockley theory [52]:

(13) $$\gamma_{g}=\gamma_{0}\theta (A-ln\theta ),$$

(14) $$\gamma_{0}=\frac{Gb}{4\pi \;(1-\nu )},$$

(15) $$A=1+ln(\frac{b}{2\pi {\;r}_{core}}),$$

where *b* is the Burgers vector (*b* = 0.2863 nm for Al), *G* is the shear modulus (*G* = 26.5 GPa for Al), ${\;r}_{core}$ is the radius of the dislocation core, which is the length that satisfies $r_{core}=\frac{3}{4}b$ [53] (in the present case ${\;r}_{core}$ = 2.15 × 10^−10^ m), *ν* Poisson’s ratio (*ν* = 0.35 for Al) and *θ* is the misorientation angle (*θ* = 15°) [19,42,52]. The *γ_g_* estimated according to Equations (13)–(15) is 0.192 Jm^−2^. In numerous literature sources, the value of grain boundary energy is claimed to be even higher (up to 0.3 Jm^−2^) [17,19].

Knowing the value of *γ_g_*, the incubation period of RX is estimated by Equation (12) and is found to be ∆t = 0.50 s for *γ_g_* = 0.192 Jm^−2^, *T* = 562 °C and *E_D_* = 2.48 × 10^5^ J/m^3^.

Another microstructural parameter, which can be derived by means of grain boundary energy and *E_D_*, is the critical radius of nuclei, which tend to develop at the early stages of recrystallization. This can be calculated with the help of Equation (4), using the corresponding stored energy value. As can be seen in Figure 8 and Figure 10, annealing for 10 s accounts for $X_{V}$ of ~5%; this implies that nucleation takes place at the very beginning of the process (according to the above calculations, Δ*t* = 0.50 s). When analyzing the release of stored energy (Figure 6), it has been noted that there is no significant drop in *E_D_* between the deformed state and the sample annealed for 10 s. Therefore, one can take the *E_D_* value characteristic of the deformed state and, in this case, the computed $r_{c}$ is 1.55 µm.

## 4. Modeling the RX Kinetics 

The analytical model enabling the description of RX kinetics is developed with the idea that the evolution of recrystallization can be approximated for different annealing temperatures and various applied strains. The experimental studies described above were conducted for the material cold-rolled with the equivalent strain (*e*) of 0.88 and subsequently annealed at 562 °C. The numerical algorithm is first applied to one straining level and particular annealing temperature, and then extended to other thermomechanical processing conditions. 

It is evident that the deformation process has a great impact on RX, since different straining conditions account for a diverse accumulation of dislocations in polycrystalline aggregates. In view of this, it is of crucial importance to estimate both the dislocation density *ρ* and stored energy *E_D_*. The numerical approach of Kubin–Estrin [28], modified by Csanádi et al. [29,30], enables the computation of dislocation density as a function of applied strain *e* with the help of the following relationship:

(16) $$\rho \left(e\right)=\frac{2C_{1}}{C_{4}}-\left(\frac{2C_{1}}{C_{4}}-\rho_{0}\right)\left(1+\frac{C_{4}e}{2}\right)\exp\left(-C_{4}e\right),$$

In Equation (16), $\rho_{0}$ is the dislocation density of unstrained material (10^10^ m^−2^ for aluminum [35]) while *C*_1_ and *C*_4_ are model parameters (for aluminum: *C*_1_ = 2.33 × 10^14^ m^−2^ and *C*_4_ = 1.15 [30,42]. In the present study, the dislocation density of 1.834 × 10^14^ m^−2^ is estimated for the Von Mises equivalent strain of 0.88. By employing Equation (6), the corresponding stored energy can be calculated and, in the given case, *E_D_* = 2.24 × 10^5^ Jm^−3^. 

The critical radii of dislocation-free nuclei, formed during RX, as well as the incubation period for the formation of nuclei, can be estimated by employing Equation (4) ($r_{c}$), (6) (*E_D_*), (12) (∆t), (16) (ρ), and (13)–(15) ($\gamma_{g}$). The *E_D_* calculated from the K–E model provides the incubation period ∆t = 0.65 s and critical radius $r_{c}$ = 1.72 µm. Once the nuclei are formed, the HAGBs will gain particular mobility due to the high temperature that is applied (see Equation (7)). The mobility of high-angle grain boundaries for *T* = 562 °C was estimated as $M_{GB}$ = 2.98 × 10^−12^ m^4^J^−1^s^−1^. Since both the residual energy of dislocations and mobility of HAGB are known, the corresponding velocity of HAGB was obtained as $G_{v}$ = 6.65 × 10^−7^ ms^−1^ employing Equation (5).

**Figure 12:**
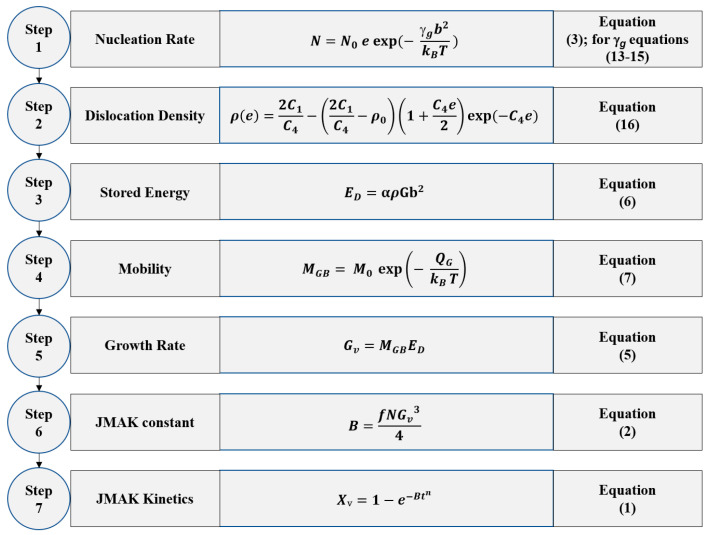
Flow chart for modeling of JMAK kinetics.

To calculate the number of nuclei generated per unit volume within one second (*N*) (see Equation (3)), it is necessary to estimate the model parameter *N*_0_. As expressed in Equation (3), the process of nucleation is directly affected by the degree of strain. In the case of the Al alloy deformed with the strain of ~1.38 and annealed at 300 °C, the deformed microstructure contained Cube bands with a length of 34.7 µm, thickness of 3.25 µm, and Cube band spacing of 26.9 µm [54]. This microstructural heterogeneity with the approximate elliptic volume of 1.27 × 10^−14^ m^3^ tended to produce at least three nuclei at low annealing temperature (*T* = 300 °C) [54]. In this case, the total number of nuclei obtained for the incubation period of 1 s is 2.36 × 10^14^ m^−3^s^−1^. In numerous instances, the deformed grains might produce up to 10 nuclei, depending on crystallographic orientation [55,56] Given this, let us consider that the microstructure of the deformed volume produces five nuclei on average, for an incubation period of 0.61 s as Δ*t* < 1 at higher temperature, i.e., *T* = 562 °C [57]. The nucleation rate is estimated to be 6.45 × 10^14^ m^−3^s^−1^ for the current case, where *e* = 0.88 and *T* = 562 °C. Knowing the total nucleation rate, one can estimate the *N*_0_ by rearranging Equation (3). The computed value of *N*_0_ = 2.87 × 10^15^ m^−3^s^−1^ is independent of strain and temperature and allows for calculation of the *B* constant for JMAK distribution by employing Equation (2). This, in turn, enables the kinetics of recrystallization to be simulated on the condition that the Avrami exponent (*n*) is known. To understand the nature of exponent *n,* the kinetics of RX are constructed, taking *n* as a fitting parameter for samples annealed 562 °C with *e* = 0.88 and 450 °C with *e* = 52 (sample D1), *e* = 70.6 (sample D2), *e* = 90.7 (sample D3). The experimental data for recrystallized fractions are estimated from the results of Vickers hardness testing using Equation (10). A systematic representation of the developed modeling method is provided in Figure 12; this is applicable to various *T* and *e* while keeping *N*_0_ = 2.87 × 10^15^ m^−3^s^−1^ constant.

**Figure 13:**
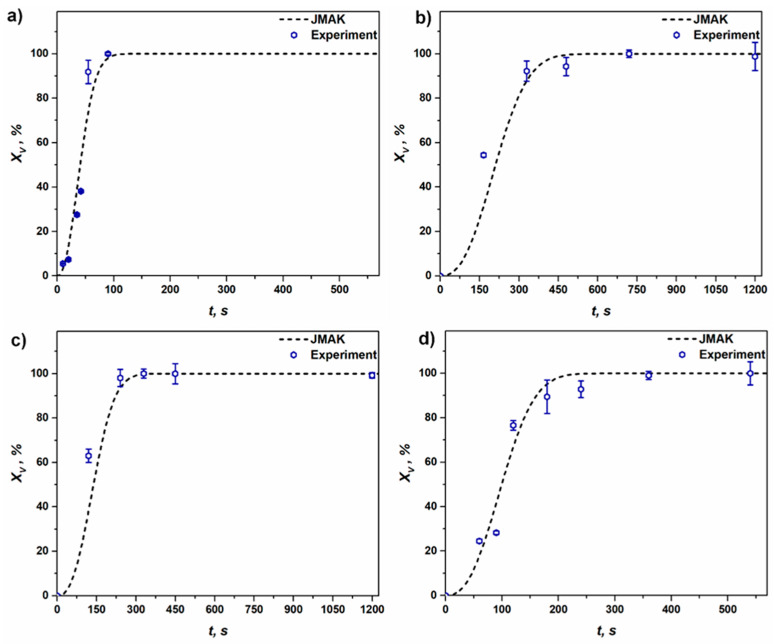
Simulated and experimentally observed kinetics of recrystallization for (**a**) initial sample, 53.35% strained and annealed at *T* = 562 °C; (**b**) sample D1, 36% strained and annealed at *T* = 450 °C; (**c**) sample D2, 46% strained and annealed at *T* = 450 °C; (**d**) sample D3, 55% strained and annealed at *T* = 450 °C (the kinetics of RX was simulated with the Avrami exponent *n* = 2.5 for *T* = 450 °C and *n* = 2.2 for *T* = 562 °C).

It can be seen from Figure 13 that the deformed sample with 53% reduction and annealed at *T* = 562 °C is fully recrystallized after ~100 s; however, in the case of samples D1, D2 and D3 annealed at *T* = 450 °C, the complete recrystallization of the deformed matrix is observed after ~600 s, ~400 s and ~300 s, respectively. At a high annealing temperature of 562 °C the kinetics of RX are faster compared to the sluggish nature of the transformation curve obtained at *T* = 450 °C for various straining ranges; however, a drop in fitting parameter *n* is observed. Along with that, the effect of straining level on RX kinetics can also be observed in Figure 13b–d; however, all the kinetics fit well with the Avrami exponent *n* = 2.5.

## 5. Discussion

The experimental studies of both microstructure and crystallographic texture suggest that the investigated material was fully recrystallized and free of strain (pre-rolled sample) prior to deformation (see Figure 2 and Figure 3). The IPF, IQ+IPF, and KAM maps presented in Figure 4 reveal all features of deformation with the presence of a complex dislocation structure (initial sample). In the deformed state, the hardness of the material is found to be greatly elevated as compared to the virgin sample, while the estimated dislocation density was 2.03 ×$\;10^{14}$ m^−2^.

While investigating the kinetics of RX, it has been observed that the Vickers hardness testing is an efficient tool to study the softening that occurs during annealing (see Figure 5). The hardness values can provide qualitative information about softening. For deformed materials with a strain exceeding *e* > 0.2, the indentation technique is very efficient in terms of predicting both dislocation density and stored energy (*E_D_*) [35,42]. However, while the same technique is used to estimate *ρ* and *E_D_*, for annealed samples, the effect of grain boundary strengthening on hardness values must be taken under consideration (see Equation (9)). This is due to the fact that indentation overestimates the dislocation density in fully recovered or slightly deformed metals [35]. Apart from that, the KAM map of the deformed material (see Figure 4) depicts the inhomogeneous distribution of dislocation statistics, causing different parts of the microstructure to relax differently during annealing; therefore, numerous indentation points are required to provide a representative value. This is particularly true for the case of partially recrystallized samples. The estimated stored energies from hardness measurements are found to be similar to those predicted by the OIM technique. It is evident that both methods are equally efficient in estimating the *E_D_*. This also validates the approach of estimating overall dislocation density considering the fact that it consists of about 50% of GND and 50% SSD (or ~2 × $\rho_{GND}$) for from moderate to highly strained materials [42]. Figure 6 indicates that the same can be true for annealed material up to a certain level of softening (in the present case *t* = 42 s). Figure 5 and Figure 6 indicate that there is an overall drop in hardness and stored energy as the RX transformation process proceeds towards its completion.

The IPF maps presented in Figure 8 show different stages of the annealing performed at 562 °C. At the early stages (samples B and C), various microstructural phenomena such as the evolution of regions with a low misorientation (precursors of nucleation) or disappearance of HAGBs are observed, while a significant fraction of the microstructure still consists of deformed grains. This can be caused by the presence of an inhomogeneous driving pressure throughout the microstructure. A similar microstructural phenomenon was reported in the literature, as well throughout the course of annealing [58]. With an increase in annealing time, recrystallization tends to progress, and a high number of recrystallized grains are observed in the deformed matrix. The impact of the annealing phenomena is directly related to the drop in both the hardness and the residual energy of dislocations in the window of annealing time between 42 s and 55 s. The distribution in grain size (shown in Figure 7) implies an increase in grain size for the fully recrystallized material from its deformed state, which could be greatly affected by both the grain size of the virgin sample and the amount of deformation. The ODF sections presented in Figure 9 show a continuous transformation from a deformed texture to its recrystallization counterpart, revealing the crystallographic nature of recrystallization. Analyzing the texture evolution (Figure 9), average Taylor factors (see Table 1), and estimated stored energies (Figure 6), one can conclude that recrystallization is driven by the low-stored energy nucleation.

To study the kinetics of RX using JMAK theory, it is important to evaluate the volume fraction of recrystallization ($X_{V}$), which can easily be obtained from the hardness value of the respective annealed samples using Equation (10). Along with that, a relatively simple method of Boltzmann fitting (Equation (11)) can also provide the transformation half-life.

While estimating various parameters of JMAK kinetics, the mobility of HAGB was recorded to be 2.98 × 10^−12^ m^4^J^−1^s^−1^, which is a function of annealing temperature. However, the velocity of HAGB is strongly affected by the annealing time and tends to slow down as the RX process approaches completion (see Figure 11). As the recrystallized grains grow at the expense of the residual energy of dislocations, the decrease in stored energy greatly impacts the kinetics by slowing down the velocity of the HAGB, as indicated by the experimental evidence. A similar relationship between $G_{v}$ and *E_D_* with an increase in annealing time was reported by Gordon and Vandermeer [59]. For the nucleation process to begin, the incubation period of Δ*t* = 0.50 s was estimated for the rolled sample exposed to annealing at 562 °C. The Δ*t* was found to be a function of annealing temperature and the degree of straining. The estimated critical radius of nuclei $r_{c}$ = 1.55 µm was expected to be in the range of 1–2 µm for 99.9% pure aluminum as per the literature [60]. The nature of $r_{c}$ is such that it increases with the decrease in stored energy. In the present study, the stored energy was found to drop with an increase in annealing time, which implies that viable nucleation is only possible at the beginning of the transformation process where the instantaneous stored energy is enough to produce fertile nucleation. However, in the present case, the incubation period was found to be as short as 0.50 s. 

The results of simulated RX kinetics (see Figure 13) show that, for the range of temperature and strain presented in this study, the fitting parameter *n* is above 2, which is an indication of the site-saturation type of nucleation, where the nucleation sites are exhausted early in the transformation process. The Avrami constant *n* (~2) derived in the present study is comparable with the vast variety of experimental evidence that claims *n* ~ 2 in the case of Al alloys [12,55,61,62,63]. As claimed by Doherty et al. [64], the practical value of *n* for omnidirectional growth is ~2, caused by both inhomogeneously distributed nucleation sites and a decrease in the velocity of HAGB as the RX process proceeds toward completion. The above assumption is also justified in the present study. As one can see in Figure 8, the tendency of the transformation of deformed grains to RX grains varies throughout the microstructure during the annealing process, which must be promoted by heterogeneously distributed favorable nucleation sites, i.e., the inhomogeneous distribution of *E_D_* throughout the microstructure. Figure 11 also clearly shows a sharp decrease in the velocity of HAGB with an increase in *t*.

The developed numerical model predicts a faster RX transformation for the annealing temperature of 562 °C, while for the *T* = 450 °C the process of RX transformation is sluggish and more time is required to complete full recrystallization (see Figure 13). In the case of samples annealed at *T* = 450 °C, it was observed that with an increase in straining level, the time required to achieve complete RX transformation decreases. This type of distinctive RX behavior is characterized by the fact that a higher straining level ensures a larger driving force for RX, which makes it possible to decrease the time needed to achieve a fully recrystallized state. When the RX kinetics of sample D3, annealed at 450 °C, and the initial sample, deformed with a 53% reduction and annealed at 562 °C, are compared, it becomes clear that although both samples accommodate a similar amount of deformation, the effect of annealing temperature is immense on RX transformation kinetics. Avrami exponent is supposed to be independent of temperature, however, in the present case, the *n* was lower for *T* = 562 °C (*n* = 2.2) compared to 450 °C (*n* = 2.5). A similar trend for a decrease in *n* was reported in other literature sources [65,66]. The nature of RX kinetics can be attributed to the appearance of precipitation during recrystallization at various temperature ranges. The influence of the precipitation behavior of iron on RX kinetics of commercial-purity aluminum is well-documented in Refs. [67,68,69]. As reported by Holm and Horbogen [68], for various annealing temperatures and strain ranges, microstructural transformations take place under the mutual influence of precipitation of iron impurities. A similar phenomenon is expected to take place in the present case, as the aluminum 1050 alloy contains ~0.3 wt% of Fe. The initial sample, annealed at 562 °C and 53.35% deformed, associated with the high amount of stored energy. The high annealing temperature and straining level/stored energy will accelerate the precipitation mechanism thereby trying to impede the progress in RX transformation. The impact of this physical phenomenon is recorded in terms of a decline in the value of Avrami exponent *n*. Similarly, a drop in mobility of HAGB is recorded, from 2.98 × 10^−12^ m^4^J^−1^s^−1^ for *T* = 562 °C to 1.01 × 10^−12^ m^4^J^−1^s^−1^ for *T* = 450 °C. This observation is consistent with the experimental observations reported by Li et al. [70].

It can be seen that the analytical approach described in the current work (see Figure 13), along with the simplified K–E model [28,29,30], can predict the JMAK kinetics at various annealing temperatures and after different degrees of deformation. A slight discrepancy between the experimental and simulated counterparts of JMAK curves was noted in Figure 13. This difference can be attributed to the heterogeneous distribution of deformation in a textured polycrystalline matrix (see Figure 4), which leads to the development of inhomogeneous recovery and RX in the early phases of annealing (see Figure 8), and the fact that the driving force of RX does not remain constant throughout the recrystallization process. The current analytical model is also able to predict the model values necessary for the simulation of RX kinetics. By comparing the RX model parameters with the experimentally reported and numerically obtained data for the same material (see Table 2), which was subjected to deformation *e* = 0.88 and subsequently annealed at 562 °C, one can notice that these values are in close proximity.

The current research work not only deals with the microstructural and textural aspects of annealing phenomena but also successfully showcased a method enabling the calculation of various parameters associated with RX, such as the mobility of high-angle grain boundaries, the velocity of-high angle grain boundaries, critical radius, and incubation period. All these parameters are obtained from the Vickers hardness and the estimated physical quantities related to recrystallization are additionally verified by various literature sources (see Table 2). On the other hand, the estimation of the above-mentioned RX parameters by adopting direct experimental techniques or modeling approaches can be an extremely tedious and complex process [19,49,71,72,73,74,75,76]. The Kubin–Estrin (K-E) model [28,29,30] is an established method to calculate the dislocation density; hence, the estimation of stored energy becomes a very simple process. When the developed algorithm (Figure 12) is applied to different straining levels and annealing temperatures, the given model can efficiently predict the kinetics of recrystallization. Hence, the algorithm of Figure 12 is not only simple but also efficient in terms of the prediction of RX kinetics. By using proper model parameters in the K-E model, the developed numerical algorithm can be extended to other metals and implemented in industrial practice.

**Table 2:** Experimental values of JMAK parameters at *T* = 562 °C with literature verification.

Variables	Reported Values	Model Value	Experimental Values (Comments)	
Mobility of HAGB *M_GB_*, m^4^J^−1^s^−1^	~2.5 × 10^−12^ [71]	2.98 × 10^−12^	2.98 × 10^−12^	Calculated by Equation (7)
Velocity of HAGB, *G_v_*, m/s	~10^−8^–10^−5^ [72,77]	6.65 × 10^−7^	7.37 × 10^−7^	Calculated by Equation (5), from hardness-based *E_D_*
Incubation Period Δ*t*, s	<1 [57]	0.65	0.50	Calculated by Equation (12), from hardness-based *E_D_*
Nucleation Rate *N*, m^−3^s^−1^	~(10^13^–10^15^) [76]	6.45 × 10^14^	-	
Critical Radius *r_c_*, µm	1–2 [60]	1.75	1.55	Calculated by Equation (4), from hardness-based *E_D_*

## 6. Conclusions

The results of the current investigation show that the indentation technique is capable of qualitatively revealing the occurrence of softening caused by recrystallization. While quantifying the release of energy stored by the annealed sample using hardness tests, the effect of grain boundary strengthening must be taken into consideration.

An analysis of microstructure evolution after different annealing stages shows that the kinetics of recrystallization can be described by the JMAK equation with the RX parameters derived either from the experimental evidence or the numerical approaches described in this study.

The numerical algorithm presented in the current investigation can accurately describe the kinetics of recrystallization at various annealing temperatures and different ranges of deformation levels. The presented simulation technique is also efficiently able to capture the effect of temperature and strain on RX kinetics while revealing the nature of Avrami exponent *n*. The model parameters computed by the presented analytical technique are in good agreement with the corresponding experimental values and those reported in various literature sources.

## Figures and Tables

**Table 1:** Taylor factor values and GNDs of initial sample and samples B–F.

Sample	Taylor Factor (M)	GND Density (m^−2^)
Initial	3.24	9.62 × 10^13^
B	3.22	5.86 × 10^13^
C	3.18	4.59 × 10^13^
D	3.23	4.28 × 10^13^
E	3.02	2.89 × 10^12^
F	3.10	-

## Data Availability

Data available on request.
